# Transcriptomics and iTRAQ-proteomics analyses provide novel insights into the defense mechanism of black shank disease in tobacco

**DOI:** 10.3389/fpls.2022.991074

**Published:** 2022-10-20

**Authors:** Ge Bai, Dun-Huang Fang, Da-Hai Yang, Zhi-Jun Tong, Xue-Jun Chen, Ming-Liang Fei, Jiu-Ling Gong, He Xie, Bing-Guang Xiao

**Affiliations:** ^1^Tobacco Breeding and Biotechnology Research Center, Yunnan Academy of Tobacco Agricultural Sciences, Kunming, China; ^2^Key Laboratory of Tobacco Biotechnological Breeding, Kunming, China; ^3^National Tobacco Genetic Engineering Research Center, Kunming, China; ^4^Lincang Company of Yunnan Tobacco Company, Lincang, China

**Keywords:** tobacco black shank, iTRAQ-proteomics, transcriptomics, PR-1B, gene expression

## Abstract

Black shank disease caused by *Phytophthora nicotianae* is one of the most important diseases in tobacco worldwide and can result in a devastating loss in tobacco cultivation. Many efforts have been carried out to identify the chromosome segment from *Nicotiana plumbaginifolia* containing a resistance locus carrying a gene named *Php*; however, the *Php* gene has not been cloned, and knowledge of the potential mechanism of the *Php* gene in the resistant lines is limited. To further characterize the resistance mechanism of the *Php* gene, we first used the resistant line “RBST” and the susceptible cultivar “Honghuadajinyuan” (HD) to obtain the near-isogenic line RBS89 containing the *Php* gene from RBST. RBS89 showed high resistance to black shank disease. Transcriptomic and iTRAQ analyses were applied to explore the potential defense mechanisms in RBS89 plants in comparison with HD plants with or without inoculation. Many differentially expressed genes (DEGs) and proteins were identified, and some pathogenesis-related (PR) proteins were extensively abundant in the RBS89 plants when compared with the HD plants in response to black shank disease. Importantly, overexpression of the PR gene *NtPR-1B* in HD plants improved the resistance of tobacco plants to black shank disease, indicating that *NtPR-1B* and *Php* genes might have similar roles in protecting tobacco from black shank disease. However, the relationship between *NtPR-1B* and *Php* genes requires further analysis. Therefore, our study provides valuable information for breeding tobacco cultivars with black shank disease resistance and sheds light on the defense mechanism of black shank disease in tobacco for enhancing *Phytophthora* resistance in other Solanaceae crops.

## Introduction

Black shank disease caused by *Phytophthora parasitica* Dast. var. *nicotianae* (Breda de Haan) Tucker is a severe disease in tobacco production worldwide, particularly in China and the United States of America (Erwin and Ribeiro, 1996; Panabieres et al., 2016; Gallup et al., 2018). The area affected by this disease is more than 76,372 ha, and the annual economic loss in China is over one billion dollars (Wang et al., 2019). *Phytophthora* not only affects tobacco but is also harmful to tomato (*Lycopersicum esculentum*) and citrus (*Citrus* spp.), and *Phytophthora parasitica* Dast. var. *nicotianae* (Breda de Haan) Tucker can infect as many as 255 species from 90 families (Cline et al., 2008; Panabieres et al., 2016). *Phytophthora parasitica* var. *nicotianae* infects the leaves, stems, and roots of tobacco at various stages, significantly affecting yield and quality (Erwin and Ribeiro, 1996; Lamour, 2013). It is difficult to prevent this disease due to their long-surviving resting structures (oospores and chlamydospores) and cryptic infection sites at an early stage. In the early stage of black shank disease infection, *P. nicotianae* obtains nutrition from plant cells and develops a necrotrophic lifestyle due to the death of plant cells (Lee and Rose, 2010; Wang, 2022). The application of chemical compounds is one method for controlling black shank disease (Ji et al., 2014; Suohui et al., 2022). However, the chemicals are not only costly but also harmful to the environment. Therefore, the utilization of resistant tobacco resources has been shown to be the most economical, effective, and environmentally friendly strategy for controlling the disease. Thus, revealing the molecular mechanisms of black shank disease in tobacco could help identify useful genetic loci for controlling and preventing black shank disease infection.

Four physiological races of *P. nicotianae* (0, 1, 2, and 3) have been defined as the ability of the pathogen to infect various cultivars carrying different resistance genes (Meng et al., 2014). Among them, race 0 is the most widely distributed and the most harmful to tobacco plants. Race 1 is less adaptable (Lucas, 1975; Speight, 1994; Sullivan et al., 2010). Races 2 and 3 have a restricted distribution and are found only in some parts of the United States, South Africa, and India (Gallup and Shew, 2010). Currently, there are two resources available in tobacco for resistance to black shank disease. Resistance to *P. nicotianae* in “Florida 301” is conferred by multiple genes, providing resistance to different races. Moreover, there are two types of resistance resources that are controlled by a single gene. One is the *Php* gene from *Nicotiana plumbaginifolia*, which has been used in flue-cured tobacco. The other is the *Phl* gene from *N. longiflora*, which has been used only in burley tobacco (Chaplin, 1962; Johnson et al., 2002a; Berbeć and Doroszewska, 2020). *Php* and *Phl* genes confer resistance to race 0 but not race 1.

Flue-cured tobacco is the most widely cultivated tobacco variety in the world, and race 0 is the most harmful to flue-cured tobacco. The tobacco resource containing the *Php* gene has been widely used for breeding resistant tobacco cultivars. Preliminary mapping-based cloning has been carried out to identify the *Php* gene (Johnson et al., 2002a,b, 2009; Bao et al., 2019); however, the *Php* gene has not been cloned until now. Therefore, unraveling the potential mechanism of the *Php* gene would benefit the development of a resistance resource for black shank disease in tobacco. A previous study revealed the resistance mechanism of the *Php* gene at the transcriptomic level (Yang et al., 2017). To further characterize the resistance mechanism of the *Php* gene, we first used the inbred line “RBST” and the susceptible cultivar “Honghuadajinyuan” (HD) to obtain the near isogenic line (NIL), RBS89. Then, transcriptomic and iTRAQ analyses were performed between RBS89 and HD plants with or without black shank inoculation, and many differentially expressed gene (DEGs) and differentially abundant proteins (DAPs) were identified. Based on iTRAQ analysis, the potential *NtPR-1B* gene was identified. Moreover, overexpression of *NtPR-1B* in HD plants improved resistance to black shank disease in tobacco, suggesting that the *NtPR-1B* gene plays an important role in enhancing tobacco defense and that *NtPR-1B* might have a similar role to the *Php* gene. These results improved our understanding of the *NtPR-1B* gene for breeding black shank disease-resistant tobacco cultivars and shed light on the defense mechanism of black shank disease in tobacco for developing *Phytophthora*-resistant cultivars in other Solanaceae crops.

## Materials and methods

### Plant materials and disease evaluations

Flue-cured tobacco (*Nicotiana tabacum*) cultivar “Coker 371-Gold” possesses a dominant gene (*Php*) originating from *N. plumbaginifolia* that confers high resistance to black shank disease caused by race 0 of the soilborne pathogen *Phytophthora parasitica* var. *nicotianae* (Johnson et al., 2002a). Coker 371-Gold was hybridized with Coker 176 to produce the F1 generation. Haploid plants were produced by crossing F1 plants (Coker 371-Gold × Coker 176) to *N. africana* (pollinator) using the maternal haploid method (Burk et al., 1979). Chromosome doubling was performed on haploid plants using the midvein culture method (Kasperbauer and Collins, 1972) to produce the flue-cured tobacco doubled haploid (DH) inbred line RBST, which is highly resistant to black shank disease. NIL RBS89 was then constructed by a hybrid cross between HD and RBST, with six generations of continuous backcrossing and one generation of selfing.

Seeds of tobacco cv. “Honghua dajinyuan” and “RBS89” were obtained from the Yunnan Academy of Tobacco Agricultural Sciences (Yunnan, China). The seeds were surface-sterilized in 40% bleach solution for 10 min, followed by three washes in distilled water, and directly sowed into the soil in pots. Young tobacco seedlings were grown in a plant growth chamber with a 16-h light/8-h dark photoperiod under continuous white light (∼75 mol m^–2^ s^–1^) at 28°C during the day and 23°C at night. All plants were well-watered after sowing.

Approximately 1 month after emergence, the seedlings were transplanted to individual cells of trays. Plants were inoculated 7 day after transplanting by first wounding the base of the plant stem with a knife. Approximately 5 g of *P. nicotianae* race 0 infested rice (*Oryza sativa* L.) grains were then added to soil near the wounded stem of each plant, and lightly covered with soil. The infested rice grains were prepared by culturing sterilized rice grains at 28–30°C for 10–14 day with a selected *P. nicotianae* isolate of Chinese origin. After the plants were inoculated, the temperature was maintained at approximately 32°C. Soil moisture was uniformly maintained by sub-irrigation. The number of dead plants for each genotype was recorded at 6 and 15 day after inoculation and used to calculate the survival ratio.

### Protein quantification and digestion

Five g stem samples were used for extracting the protein by 10% TCA/50% acetone, and 300 μg of proteins of each samples were diluted in 4% SDS, 100 mM Tris-HCl (pH 8.0). The protein heated at 100°C for 5 min. Each sample was then cooled to room temperature and loaded onto an ultrafiltration filter (30 kDa cutoff, Sartorius, Germany) containing 200 μl of UA buffer (8 M urea, 150 mM Tris-HCl, pH 8.0), followed by centrifugation at 14,000 × *g* for 30 min and an additional washing step with 200 μl of UT buffer. One hundred microliters of 50 mm iodoacetamide in UA buffer was subsequently added to the filter to block the reduced cysteine residues, and the samples were incubated for 30 min at room temperature in the dark, followed by centrifugation at 14,000 × *g* for 30 min. The filters were washed three times with 100 μl of UA buffer and centrifuged at 14,000 × *g* for 30 min after each washing step. Subsequently, 100 μl of dissolution buffer (Applied Biosystems, Foster City, CA, United States) was added to each filter, followed by centrifugation at 14,000 × *g* for 30 min, which was repeated three times. The protein suspensions were then digested with 40 μl of trypsin (Promega, Madison, WI, United States) buffer (6 μg trypsin in 40 μl dissolution buffer) at 37°C for 18 h. Finally, the filter unit was transferred to a new tube, and 40 μl dissolution buffer was added and centrifuged at 14,000 × *g* for 30 min. The resulting peptides were collected as a filtrate, and the peptide concentration was analyzed at OD_280_.

### iTRAQ labeling and fractionation

iTRAQ labeling was performed according to the manufacturer’s instructions (Applied Biosystems, United States). Briefly, the peptide mixtures were reconstituted with 30 μl of iTRAQ dissolution buffer. The label method was performed for each sample (100 μg) using the iTRAQ Reagent-8plex Multiplex Kit (AB SCIEX, United States). The labeling solution reaction was then incubated at room temperature for 1 h prior to further analysis. The six labeled samples were pooled into one sample and dried in a vacuum centrifuge at room temperature. The iTRAQ-labeled peptides were subjected to High-pH Reversed-Phase Fractionation in a 1100 Series HPLC Value System (Agilent, United States) equipped with a Gemini-NX (Phenomenex, 00F-4453-E0) column (4.6 mm × 150 mm, 3 μm, 110 Å). The peptides were eluted at a flow rate of 0.8 ml/min. Buffer A consisted of 10 mm ammonium acetate (pH 10.0), and buffer B consisted of 10 mm ammonium acetate and 90% v/v CAN (pH 10.0). The following gradient was applied to perform separation: 100% buffer A for 40 min, 0–5% buffer B for 3 min, 5–35% buffer B for 30 min, 35–70% buffer B for 10 min, 70–75% buffer B for 10 min, 75–100% buffer B for 7 min, 100% buffer B for 15 min, and 100% buffer A for 15 min. The elution process was monitored by measuring and was finally combined into 15 pools. Each fraction was concentrated *via* vacuum centrifugation and reconstituted in 40 μl of 0.1% v/v trifluoroacetic acid. All samples were stored at −80°C until LC-MS/MS analysis.

### LC-MS analysis

A total of 1 μg of each sample was loaded onto a Thermo Scientific EASY column (two columns) using an autosampler at a flow rate of 200 ml/min. The sequential separation of peptides on a Thermo Scientific EASY trap column (100 μm × 2 cm, 5 μm, 100 Å, C18) and analytical column (75 μm × 25 cm, 5 μm, 100 Å, C18) was accomplished using a segmented 1 h gradient from 5 to 28% solvent B (0.1% formic acid in 100% ACN) for 40 min, followed by 28–90% solvent B for 2 min and then 90% solvent B for 18 min. The column was re-equilibrated to its initial highly aqueous solvent composition before each analysis. The mass spectrometer was operated in positive ion mode, and MS spectra were acquired over a range of 350–2,000 m/z. The resolving powers of the MS scan and MS/MS scan at 100 m/z for the Orbitrap Elite were set at 60,000 and 15,000, respectively. The 16 most intense signals in the acquired MS spectra were selected for further MS/MS analysis. The isolation window was 2 m/z, and ions were fragmented through higher-energy collisional dissociation with normalized collision energies of 35 eV. The maximum ion injection times were set at 10 ms for the survey scan and 100 ms for the MS/MS scans, and the automatic gain control target values for the full scan modes were set to 1^*e*6^ and 5^*e*4^ for MS/MS. The dynamic exclusion duration was 30 s.

### iTRAQ data analysis

The raw files were analyzed using Proteome Discoverer 2.1 software (Thermo Fisher Scientific, United States). The fragmentation spectra were searched against P17032_NCBI_Nicotiana_tabacum_91636.fasta using the MASCOT search engine embedded in Proteome Discoverer. The following search parameters were used: monoisotopic mass, trypsin as the cleavage enzyme, two missed cleavages, iTRAQ labeling, and carbamidomethylation of cysteine as fixed modifications, peptide charges of 2^+^, 3^+^, and 4^+^, and the oxidation of methionine as variable modifications. The mass tolerance was set to 20 ppm for the precursor ions and 0.1 Da for the fragment ions. The results were filtered based on a false discovery rate (FDR) of no more than 1%. The relative quantitative analysis of the proteins in the samples based on the ratios of iTRAQ reporter ions from all unique peptides representing each protein was performed using Proteome Discoverer (version 2.1). The relative peak intensities of the iTRAQ reporter ions released in each of the MS/MS spectra were used. The final ratios obtained from the relative protein quantifications were then normalized based on the median average protein quantification ratio. Protein ratios represent the median of the unique peptides of the protein.

### Plasmid construction and tobacco transgenic plants

Total RNA was purified from tobacco leaves, and cDNA was obtained using an RNA isolation kit (Qiagen, Hilden, Germany). The full-length coding sequence of the *pathogenesis-related protein 1B* gene was amplified and cloned into the pDONR-zeo vector by a BP reaction (Invitrogen, United States), and then into pB2GW7 by an LR reaction (Invitrogen, Waltham, MA, United States). The pB2GW7, containing the *pathogenesis-related protein 1B* gene, was transformed into tobacco leaves *via Agrobacterium*-mediated transformation.

### Sample collection and library preparation

The stems, with three biological replicate samples, were used for RNA extraction using a TRIzol reagent (Invitrogen Corp., Carlsbad, CA, United States). RNA purifications were performed using an RNeasy Mini Kit (Qiagen, Chatsworth, CA, United States). Library preparation was carried out according to the Illumina Hiseq RNA sample preparation kit (Illumina, San Diego, CA, United States). All original data were deposited in the National Genome Data Center (NGDC, accession number: PRJCA010360).

### RNA-seq data processing and detection of differentially expressed genes

Raw data in FASTQ format were first processed using the NGS QC Toolkit32. In this step, clean reads were obtained by removing reads containing the adapter, reads containing ploy-N, and low-quality reads from the raw data. All downstream analyses were based on these clean data. Reads were mapped to the tobacco genome (GCA_002210045.1) using bowtie2 (version 2.4.4) (Langmead and Salzberg, 2012). The FPKM value was acquired by quantitative analysis of transcripts with RSEM (RSEM: accurate transcript quantification from RNA-seq data with or without a reference genome). Briefly, clean data were mapped back to the assembled transcriptome, and the gene expression matrix was constructed using DESeq2.^1^ The *P*-value and log_2_FC were measured using Trinity (version 2.11.0). A *P* < 0.05 and absolute value of Log_2_Foldchange ≥ 1 were adopted as the screening criteria, and the genes that fulfilled this criterion were defined as DEGs. Corresponding Venn diagrams and volcano diagrams were generated with the OriginPro 2021 software.^2^ The correlation coefficients among the samples were also calculated using the Spearman approach. The heatmaps for DEGs were conducted using the “pheatmap” package implemented in R.^3^

### Gene ontology and kyoto encyclopedia of genes and genomes enrichment analyses

The gene ontology (GO) and kyoto encyclopedia of genes and genomes (KEGG) of the DEGs were conducted with eggnog-mapper (Huerta-Cepas et al., 2017). Thereafter, we conducted functional categorization and calculated the quantities of annotated differential genes with Tbtools (Chen et al., 2020). They were eventually visualized in R version 4.2.0.

### Quantitative real-time PCR of selected genes

A total of 2 μg total RNA in a 20 μl reaction was converted to cDNA with SuperScript III Reverse Transcriptase (Invitrogen, United States) according to the manufacturer’s instructions on an Eppendorf Mastercycler thermocycler (Eppendorf AG, Germany) with the following conditions: 25°C for 5 min, 50°C for 60 min, and 70°C for 15 min, followed by a hold at 4°C until use in a Quantitative real-time PCR (qPCR) reaction. A total of 60 μl of deionized water was added to 20 μl cDNA, and 1 μl of diluted cDNA mixture was used as the input for the qPCR reaction. The qPCR reactions were performed with a SuperReal PreMix Plus SYBR Green Kit (TIANGEN Biotech, Beijing, China), following the manufacturer’s instructions in a 20 μl volume. The qPCR was performed on an Applied Biosystems™ QuantStudio™ 6 Flex Real-Time PCR System (Thermo Fisher Scientific, Waltham, MA, United States) with the following cycling conditions: 95°C for 15 min, followed by 40 cycles of 95°C for 10 s, 60°C for 20 s, and 72°C for 32 s. The melt curve conditions were 95°C for 15 s, 60°C for 1 min, and 95°C for 15 s. All samples had only one melt temperature peak. The Log_2_Foldchange was calculated using the 2^–ΔΔ*CT*^ method with 26S as a reference gene. The CT values represent the average of the three technical replicates. The sequences of primers used for qRT-PCR are listed in Supplementary Table 4.

## Results

### Tobacco NIL line RBS89 is resistant to black shank disease

**FIGURE 1:**
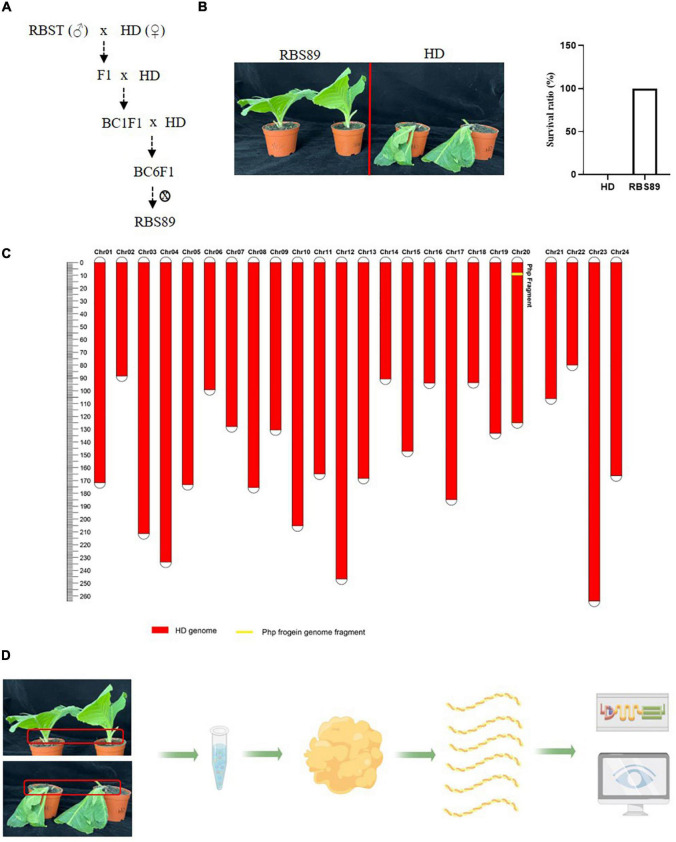
The near isogenic line (NIL) RBS89 is resistant to black shank disease in tobacco. **(A)** Construction of the RBS89 line in tobacco by six generations of contiguous backcrossing and one generation of selfing. A black shank disease-resistant line RBST was used as the donor, and black shank disease-susceptible cultivar Honghuadajinyuan (*N. tabacum*, HD) was used as the recipient. **(B)** HD and RBS89 plants were inoculated with *P. nicotianae*-infested rice, and the survival ratio was calculated. **(C)** A physical map of the NIL was constructed with molecular markers. The red areas indicate regions that were homozygous for HD alleles, and the yellow areas indicate regions homozygous for one segment introgressed from the *N. plumbaginifolia* chromosome. **(D)** Comparative analysis of HD and RBS89 plants with or without black shank disease infection by integration of transcriptomics and iTRAQ-Proteomics in tobacco.

The flue-cured tobacco cultivar HD is extremely susceptible to black shank disease. To identify the potential *Php* gene in tobacco, an NIL was constructed following hybridization between susceptible HD and resistant inbred line RBST, which contains the *Php* gene introgressed from *N. plumbaginifolia*. After six generations of continuous backcrossing and one generation of selfing, NIL RBS89 containing the *Php* gene was obtained and was highly resistant to black shank disease (Figures 1A,B). Genetic analysis showed that the gene recovery rate was up to 99.9925%, which avoided the effect of genetic backgrounds from Coker 371-GOLD for further analysis (Figure 1C).

### Transcriptomic analyses of the tobacco RBS89 line in response to *Phytophthora nicotianae*

**FIGURE 2:**
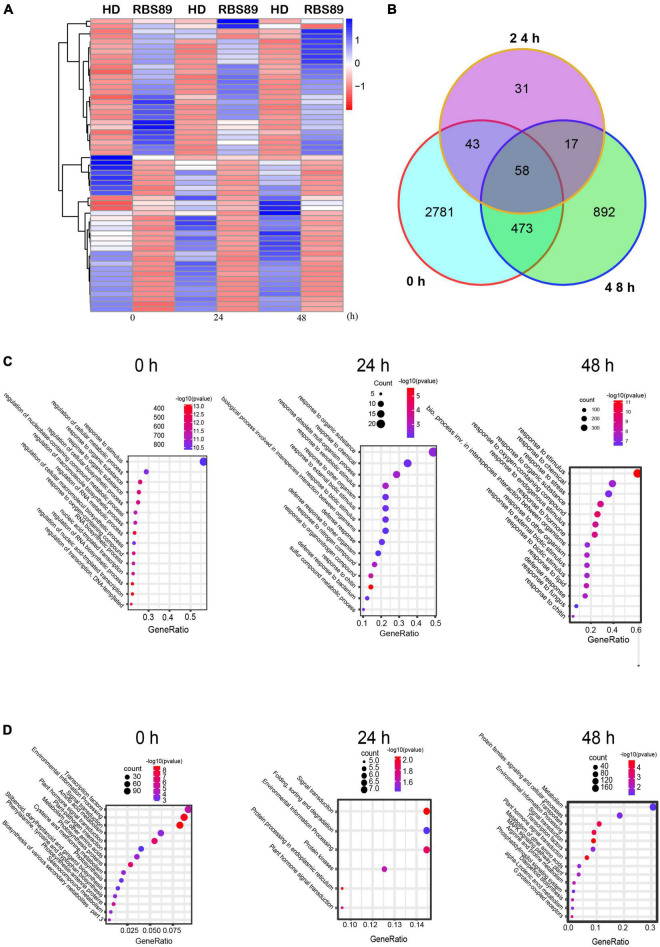
Differentially expressed genes (DEGs) of HD and RBS89 plants with or without inoculation identified from RNA-seq data. **(A)** Heatmap showing the upregulated or downregulated DEGs between HD and RBS89 plants with or without inoculation. The scaling of each gene was to a mean of 0 and a standard deviation of 2.0. **(B)** Venn diagram analysis showed 4,295 DEGs between the HD and RBS89 plants with or without inoculation. **(C)** GO enrichment analysis of the DEGs. **(D)** KEGG pathway analysis of the DEGs.

As RBS89 contained the *Php* gene, susceptible HD and RBS89 plants were inoculated with *P. nicotianae*-infested rice (*Oryza sativa* L.) grains to elucidate the potential mechanism of the *Php* gene. The stem samples with three biological repeats were harvested at 0, 24, and 48 h after inoculation, and the RNAs of these samples were subjected to high-throughput sequencing to detect DEGs (Figure 1D). A total of 47 million sequenced reads were obtained from 18 samples using Illumina RNA-seq, and almost 63% of these reads were uniquely mapped to the tobacco reference genome (Supplementary Table 1). Many DEGs (fold-change ≥ 2 and false discovery rate ≤ 0.05) were identified between the susceptible HD and RBS89 plants in response to *P. nicotianae* using DESeq2 software (Figure 2). Before inoculation, there were 3,355 DEGs (1,814 upregulated and 1,541 downregulated) in the RBS89 plants compared to the HD plants (Figure 2A and Supplementary Table 2). Compared with HD plants, 151 DEGs (87 upregulated and 64 downregulated) were detected in the RBS89 plants 24 h after inoculation, and 1,441 DEGs (438 upregulated and 1,003 downregulated) were detected 48 h after inoculation (Supplementary Table 2). A Venn diagram showed the common and individual DEGs between HD and RBS89 plants with or without inoculation for the indicated time (Figure 2B). These results suggest that many genes were differentially expressed between HD and RBS89 plants with or without inoculation, and those DEGs might contain potential candidate genes that work downstream of the *Php* gene.

To understand the functions of the DEGs that were identified between HD and RBS89 plants in response to *P. nicotianae*, gene ontology (GO) analysis was then conducted. Several functional categories were over-represented between HD and RBS89 plants with or without inoculation, including “Response to stimulus,” “Response to chemical,” “Response to organic substance,” “Response to oxygen-containing compound,” and “Response to chitin” (Figure 2C). We further identified the biological pathway of DEGs between HD and RBS89 plants with or without inoculation, and KEGG analysis was performed using DAVID with a cut-off probability ≥ 0.95 and a fold change ≥ 2. These DEGs were enriched in 27 different functional pathway categories, and 14 categories overlapped among the pairwise comparisons (Figure 2D). For example, “Transcription factors,” “Signal transduction,” “Environmental information process,” and “Plant signal transduction” were enriched between HD and RBS89 plants with or without inoculation. Interestingly, the pathway of “Plant-pathogen interaction” was also enriched between HD and RBS89 plants without inoculation (Figure 2D), indicating that these DEGs might be potential candidate genes, which might be critical factors related to the *Php* gene.

### iTRAQ-proteomics analyses of the tobacco RBS89 line in response to *Phytophthora nicotianae*

**FIGURE 3:**
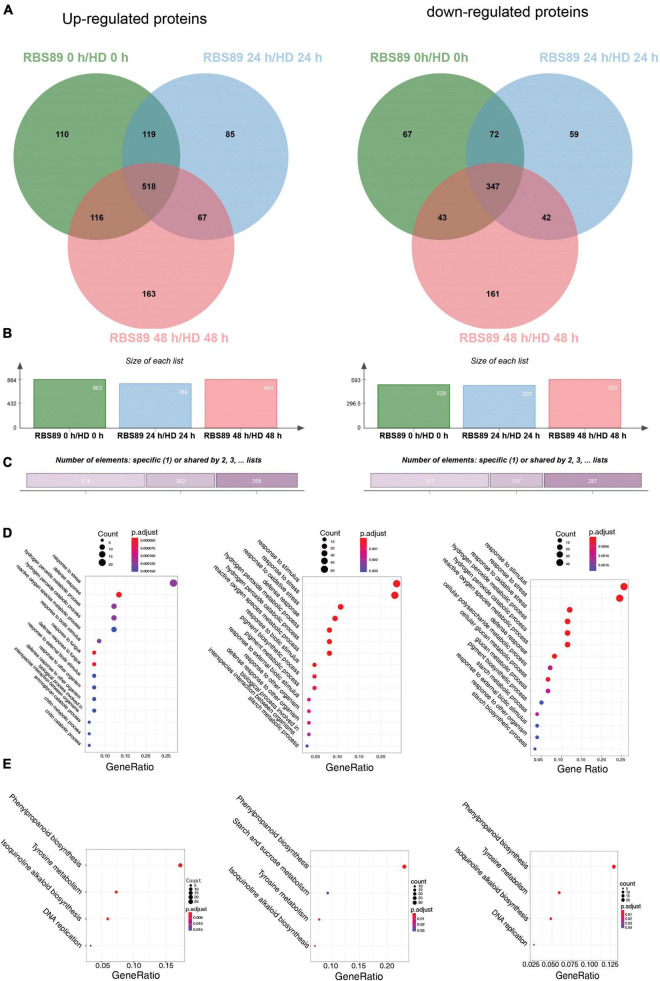
Differentially expressed proteins (DAPs) of HD and RBS89 plants with or without inoculation identified from iTRAQ data. **(A)** Venn diagram analysis showing the upregulated or downregulated proteins identified between HD and RBS89 plants with or without inoculation. **(B)** The statical number of upregulated or downregulated proteins in penal. **(C)** The upregulated (Left panel) and downregulated (Right panel) specific or shared DAPs in Figure 3A. **(D)** GO enrichment analysis of the DAPs (Left panel, 0 h; Middle panel, 24 h; Right panel, 48 h). **(E)** KEGG pathway analysis of the DAPs (Left panel, 0 h; Middle panel, 24 h; Right panel, 48 h).

To characterize the potential mechanism of the *Php* gene at the proteomic level, the above stem samples, with three biological repeats, were extracted for proteins detected by iTRAQ. A total of 27,329 peptides were obtained and annotated as 7,795 unique proteins after analysis using Mascot software (Supplementary Table 3). DAPs were then identified between HD and RBS89 at the time points with or without inoculation (Figure 3). Compared with HD plants, there were 863 upregulated and 529 downregulated proteins in the RBS89 plants without inoculation (Figures 3A,B and Supplementary Table 3). After inoculation for 24 h, a total of 789 and 520 proteins were upregulated and downregulated, respectively, in the RBS89 plants when compared with HD plants at 24 h after inoculation (Figure 3C and Supplementary Table 3). At 48 h after inoculation, there were 864 upregulated and 593 downregulated proteins in the RBS89 plants, in contrast to HD plants (Figures 3A,B and Supplementary Table 3). The Venn diagram showed that there were 865 overlapping proteins, including 518 upregulated proteins and 347 downregulated proteins, among the time points (Figure 3C), indicating that these common DAPs were stable in the RBS89 plants with or without inoculation.

To determine the biological functions of DAPs between HD and RBS89 plants in response to *P. nicotianae*, we performed a GO enrichment analysis of DAPs at these three time points. According to the biological process, molecular function, and cellular component, the proportions of enriched GO terms for DAPs were shown using the *P*-value (*P* < 0.01) (Figure 3D). Without inoculation, the DAPs were mainly enriched in the categories of response to stress, defense response, and hydrogen peroxide metabolic process (Figure 3D). At 24 h after inoculation, the enriched DAPs participated in the response to stimulus, response to stress, response to oxidative stress, hydrogen peroxide metabolic process, and hydrogen peroxide catabolic process (Figure 3D). At 48 h after inoculation, the enriched DAPs were mainly involved in the response to stimulus, response to stress, response to oxidative stress, and hydrogen peroxide metabolic processes (Figure 3D).

Moreover, the number of DAPs in different pathways varied as the time after inoculation increased. For example, there were 22, 45, and 54 DAPs in the response to the stress category at 0, 24, and 48 h after inoculation, respectively (Figure 3D). In the defense response category, there were 15, 11, and 22 DAPs at 0, 24, and 48 h after inoculation, respectively (Figure 3D). Interestingly, the number of DAPs increased in response to *P. nicotianae* in the category of response to stimulus and response to stress, there were 47 and 55 DAPs that identified after inoculation between HD and RBS89 plants, respectively, while 23 DAPs were identified without inoculation (Figure 3D). We then identified the biological pathway of DAPs between HD and RBS89 plants in response to *P. nicotianae* using KEGG (Figure 3E). In total, all DAPs were enriched in five functional pathways (Figure 3E), such as “Phenylpropanoid biosynthesis,” “Starch and sucrose metabolism,” “Tyrosine metabolism,” “Isoquinoline alkaloid biosynthesis,” and “DNA replication,” indicating that these DEP pathways might be involved in the regulation of the *Php* gene in RBS89 plants.

### Correlation analysis of differentially expressed genes and differentially abundant proteins identified by transcriptome and proteome analyses

**FIGURE 4:**
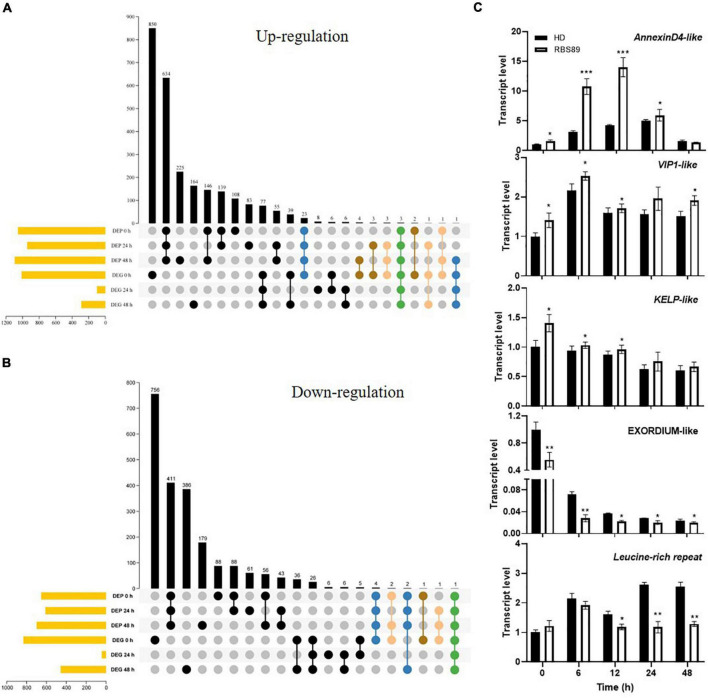
Correlation analysis of DEGs and DAPs identified by transcriptome and proteome analyses. **(A,B)** Venn diagram of the overlapping DEGs and DAPs between HD and RBS89 plants, with or without inoculation. **(C)** Validation of DEP and DEG coordinate pairs, which were pathogenesis-related proteins identified using qRT-PCR. qRT-PCR analysis was performed for five pathogenesis-related proteins identified in the correlation analysis of DEGs and DAPs between HD and RBS89 plants with or without inoculation. The expression of *Nt26S* was used as the internal control. *P*-values were calculated using a two-tailed Student’s *t*-test. Error bar means ± SD (*n* = 3). Asterisks indicate significant differences (*P < 0.05, **P < 0.01, ***P < 0.001).

Integrated transcriptome and proteome analyses were performed to identify overlapping genes between HD and RBS89 plants in response to *P. nicotianae*. Among 4,523 genes identified with transcript and protein expression, only 11 and 41 genes showed coordinated upregulated or downregulated expression patterns at both mRNA and protein levels (Figures 4A,B). Some coordinate gene/protein pairs might have a role in plant defense against pathogen invasion. For example, pathogenesis-related proteins STH-2-like, RNA polymerase II transcriptional coactivator KELP-like, transcription factor VIP1-like, and chloride channel protein CLC-c-like genes were upregulated in pathogen defense, while ethylene-responsive transcription factor 1B-like, cysteine-rich repeat secretory protein 12-like, leucine-rich repeat extensin-like protein 4, and protein EXORDIUM-like were downregulated (Supplementary Tables 2, 3).

To validate the DEGs and DAPs identified by RNA-seq and iTRAQ analysis, qRT-PCR was performed on these five overlapping genes between HD and RBS89 plants in response to *P. nicotianae*. The same expression tendency was observed between the RNA-seq and iTRAQ results (Figure 4C), indicating that these genes might participate in the regulation of the *Php* gene in tobacco’s response to *P. nicotianae*.

### Overexpression of the *PR-1B* gene improved the resistance of tobacco plants in response to *Phytophthora nicotianae*

**FIGURE 5:**
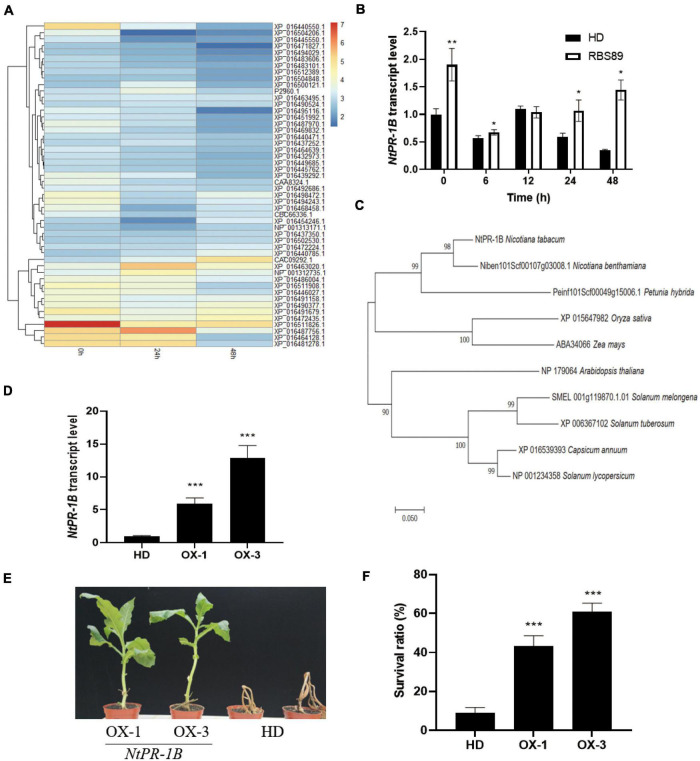
Overexpression of *NtPR-1B* improved the resistance of tobacco plants to *P. nicotianae*. **(A)** Heatmap showing the 50 most abundant proteins according to the protein expression levels in the iTRAQ analysis. **(B)** Expression of *NtPR-1B* in HD plants with or without inoculation at the indicated time, as determined by qRT-PCR. **(C)** Phylogenetic analysis of the *PR-1B* gene in Solanaceae crops **(D)** Expression of *NtPR-1B* in two overexpressors of transgenic HD plants using qRT-PCR. **(E)** Represented image of two overexpressors and HD plants in response to *P. nicotianae*. **(F)** Survival ratio of two overexpressors and HD plants in response to *P. nicotianae*. The expression of *Nt26S* was used as the internal control. *P*-values were calculated using a two-tailed Student’s *t*-test. Error bar means ± SD (*n* = 3). Asterisks indicate significant differences (*P < 0.05, **P < 0.01, ***P < 0.001).

Given that there was less overlap between mRNA and proteins, we focused on the iTRAQ results. The heatmap was built among the 50 most abundant proteins according to the protein expression levels. These 50 proteins were divided into two groups (Figure 5A). One group of 14 proteins showed relatively higher expression, and the other group of 36 proteins presented relatively lower expression (Figure 5A and Supplementary Table 3). The proteins with higher expression mainly included peroxidase 12-like, pathogenesis-related protein 1B (PR-1B), pathogenesis-related protein PR-4B, aspartic proteinase-like, pathogenesis-related protein PR-4A, polyphenol oxidase E, granule-bound starch synthase 1, quinolinate synthase, kirola-like, and acidic endochitinase P.

Interestingly, among the 14 proteins with high expression levels, three were pathogenesis-related proteins (Supplementary Table 3). Pathogenesis-related proteins have shown a wide range of disease-resistance functions. Before inoculation, three pathogenesis-related proteins (PR-1B, PR-4B, and PR-4A) showed higher expression levels, suggesting their general expression patterns (Supplementary Table 3). Compared with PR-4B and PR-4A, the expression of PR-1B was induced 24 h after inoculation and decreased at 48 h, and the expression levels of PR-1B proteins were higher than those of the other two pathogenesis-related proteins (PR-4B and PR-4A) at the same stage (Supplementary Table 3). Meanwhile, the expression levels of the *PR-1B* gene were higher in the RBS89 lines without inoculation, and with prolonged inoculation, the expression levels of the *PR-1B* gene were higher in the RBS89 lines when compared with HD plants (Figure 5B).

Therefore, we speculated that the PR-1B protein might play an important role in tobacco defense against black shank disease. In addition, phylogenetic analysis of PR-1B revealed their conservation in *Solanaceae* crops (Figure 5C). To confirm the role of the PR-1B protein, the full-length *PR-1B* gene was cloned from the RBS89 plants, and two overexpression lines were obtained from the transgenic HD plants (Figure 5D). Two overexpression lines and HD plants were grown in the greenhouse under the same conditions after being sown for 1 month. The plants were inoculated with race 0, and the HD plants were wilted 7 days after inoculation, while two overexpression lines partially survived, showing the obvious inhibition of infection in tissue necrosis at the inoculation site (Figures 5E,F) and indicating that overexpression of the *PR-1B* gene in tobacco improved resistance to black shank disease.

## Discussion

Tobacco is the most well-studied model plant and is widely used to investigate the interaction between pathogens and plants. Currently, tobacco black shank disease caused by pathogenic oomycete, *P. nicotianae*, has become one of the most devastating diseases in tobacco production. Flue-cured tobacco could not withstand pathogen infection, which resulted in discontinued growth. Tobacco black shank disease mainly affects the stem base in the field, and the risk and severity of tobacco black shank disease cause significant economic losses each year. Several chemical compounds have been used to control the disease; however, utilization of host resistance and exploration of defense mechanisms are beneficial in controlling and preventing the spread of disease in tobacco. Previous studies revealed that the resistant tobacco resource for black shank disease contained the potential *Php* gene, which was not identified (Johnson et al., 2002a,b, 2009; Bao et al., 2019), and regulation of the *Php* gene in the *Php*-containing materials was determined at the transcript level (Yang et al., 2017). Therefore, identifying the potential regulatory proteins in *Php*-containing materials should provide more detailed information on the defense mechanism of the *Php* gene.

To identify the potential *Php* gene in tobacco, NIL RBS89 was obtained, and transcriptomics and iTRAQ-proteomics analyses were performed between HD and RBS89 plants in response to *P. nicotianae*. There were many DEGs between HD and RBS89 plants with or without inoculation at different time points after inoculation (Figure 2), and the pathway of “plant-pathogen interaction” was enriched (Figure 2C), indicating that these DEGs have a potential role in the regulation of the *Php* gene for disease antagonists in tobacco. Moreover, there were higher protein levels in RBS89 plants than in HD plants in response to *P. nicotianae* in the GO analysis of the DAPs (Figure 3). The enriched DAPs belonged to the pathway of response to stress, response to stimulus, and response to oxidative stress between HD and RBS89 plants (Figure 3D), indicating that these proteins might play key roles in plant resistance to infections from pathogens, which resulted in the improvement of plant resistance in RBS89 plants. Furthermore, correlation analysis of DEGs and DAPs identified using transcriptome and proteome analysis showed that several pathogenesis-related proteins overlapped between mRNA and protein levels (Figures 4A,B) and that those coordinate gene/protein pairs might have potential roles in plant defense against pathogen invasion. However, only 52 genes that showed coordinate gene/protein pairs among the 4,523 genes in the DEGs and DAPs between the HD and RBS89 plants were identified (Figures 4A,B).

Three pathogenesis-related proteins, NtPR-1B, NtPR-4B, and NtPR-4A, showed higher accumulation at protein levels in the RBS89 plants compared with the HD plants (Figure 5A). As early as 1970, PR-1 proteins were found in TMV-infected tobacco (Van Loon and Van Kammen, 1970). Some members of the PR-1 proteins are induced by TMV infection. The PR-1 protein is one of the most abundant proteins produced in the defense response process, accounting for 2% of the total leaf proteins in tobacco infected by pathogens (Alexander et al., 1993). Overexpression of the *PR-1* gene can enhance plant resistance to fungi, oomycetes, and bacteria (Sarowar et al., 2005; Kiba et al., 2007; Shin et al., 2014). PR-4B can inhibit the growth of some pathogenic fungi in wheat (Caruso et al., 2001) and participate in defensive responses against necrotizing vegetative pathogens in *Arabidopsis* (Caruso et al., 2001). In pepper, the PR-4B protein can increase its disease resistance by promoting cell necrosis (Hwang et al., 2014). The PR-4 protein can be induced by TMV infection in tobacco (Hwang et al., 2014). Furthermore, the expression levels of *NtPR-1B* were higher than those of the other two pathogenesis-related proteins at the same treatment time (Supplementary Table 2). Therefore, we suspected that the NtPR-1B protein might play an important role in tobacco defense against black shank disease. Undoubtedly, overexpression of *NtPR-1B* inhibited infection, as observed by tissue necrosis at the inoculation site in tobacco (Figures 5E,F), implying that the NtPR-1B protein plays a critical role in tobacco’s response to black shank disease.

In general, plants have evolved an immune system of two layers, pattern-triggered immunity (PTI) and effector-triggered immunity (ETI), to respond to infections from various microbes (Jones and Dangl, 2006; Boller and He, 2009; Zhou and Zhang, 2020). With the major immune receptors on the cell surface and inside the cell, plants can recognize pathogens (bacteria, fungi, oomycetes) and initiate an immune response to protect themselves. When infected by pathogens, plants can rapidly recognize pathogen-associated molecular patterns (PAMPs) through pattern-recognition receptors (PRRs) on the surface of the cell membrane (Thulasi Devendrakumar et al., 2018) and activate the receptor-like cytoplasmic kinases (RLCKs) through binding to and phosphorylating the co-receptors (Zhang et al., 2010; Shi et al., 2013), which then activate the pathways of mitogen-activated protein kinases (MAPKs) and calcium-dependent protein kinases (CDPKs), leading to calcium ion influx and a burst of reactive oxygen species (ROS). These reactions are collectively referred to as pattern-triggered immunity (PTI) responses (Katagiri and Tsuda, 2010; Xin et al., 2018). PTI is a broad-spectrum, non-specific immune response (Katagiri and Tsuda, 2010). PTI plays an important role in reducing pathogen invasion (Thulasi Devendrakumar et al., 2018) and maintaining the homeostasis of endophytic leaf microflora in plant leaves (Thulasi Devendrakumar et al., 2018). To avoid plant PTI, the pathogen can secrete effectors to the host cells to make plants susceptible (Jones and Dangl, 2006; Xin et al., 2018). The stronger response of effector-triggered immunity (ETI) is triggered when plants can overcome PTI (Jones and Dangl, 2006). ETI can directly or indirectly recognize the effectors of the pathogen through intracellular nucleotide-binding sites and leucine-rich repeat domain receptors (NLRs), which trigger a defense response.

It is well-known that pathogenesis-related proteins act as weapons against various bacterial pathogens and provide enhanced disease resistance. Overexpression of the *PR-1B* gene can inhibit mycelial growth, which has been found in rice and tobacco (Sarowar et al., 2005; Kiba et al., 2007; Shin et al., 2014). PR-10 shows a broad spectrum of antibacterial activity against *Pseudomonas syringae*, *Agrobacterium tumefaciens*, *A. radiobacter*, *Pseudomonas aureofaciens*, and *Serratia marcescens* (Fernandes et al., 2013). Overexpression of the *PR-14* gene in rice plants showed increased resistance to bacterial and fungal pathogens (Patkar and Chattoo, 2006). Therefore, pathogenesis-related proteins participated in improving host resistance against several stressors, and an in-depth analysis of promising PR protein candidates provided insights into developing stress-tolerant crop varieties.

Collectively, we performed integrated transcriptomic and iTRAQ analyses to elucidate the resistance mechanisms of the *Php* gene in RBS89 plants and identified many DEGs and DAPs between HD and RBS89 plants in response to *P. nicotianae*. Many of these differentiated genes/proteins belonged to different signaling pathways and metabolism processes. Furthermore, there were overlapping and distinct roles for DEGs and DAPs, which might have common or specific roles in the regulation of the *Php* gene in tobacco’s response to black shank disease. Importantly, the pathogenesis-related gene *NtPR-1B* has been identified as a key factor responsible for resistance to black shank disease in tobacco. Therefore, our findings can provide valuable information for breeding tobacco cultivars resistant to black shank disease and other Solanaceae crops with *Phytophthora* resistance.

## Data availability statement

The data presented in this study are deposited in the repository of National Genomics Data Center, accession number: PRJCA010360, available at https://ngdc.cncb.ac.cn/search/?dbId=bioproject&amp;q=PRJCA010360.

## Author contributions

GB, HX, and B-GX: conceptualization. GB, HX, D-HF, and M-LF: methodology. GB, HX, and M-LF: software. M-LF, Z-JT, and X-JC: validation. GB, HX, J-LG, and D-HY: formal analysis. GB, HX, D-HF, and D-HY: writing – original draft preparation. GB, HX, B-GX, and D-HY: writing – review and editing. B-GX and HX: supervision. All authors contributed to the article and approved the submitted version.

## Footnotes

1
https://bioconductor.org/packages/release/bioc/html/DESeq2.html

2
https://www.originlab.com/2021

3
https://cran.r-project.org/web/packages/pheatmap/index.html
